# Ayurvedic preparations of *Raudra Rasa* inhibit agonist‐mediated platelet activation and restrict thrombogenicity without affecting cell viability

**DOI:** 10.1002/2211-5463.13713

**Published:** 2023-11-21

**Authors:** Susheel Nidhi Chaurasia, Vipin Singh, Mohammad Ekhlak, Manoj Kumar Dash, Namrata Joshi, Debabrata Dash

**Affiliations:** ^1^ Center for Advanced Research on Platelet Signaling and Thrombosis Biology, Department of Biochemistry, Institute of Medical Sciences Banaras Hindu University Varanasi India; ^2^ Department of Rasa Shastra & B Kalpana Government Ayurved College Raipur India; ^3^ Department of Rasa Shastra, Faculty of Ayurveda, Institute of Medical Sciences Banaras Hindu University Varanasi India

**Keywords:** AKT, CRR, mROS, MRR, platelets, RRE

## Abstract

Ayurveda is considered to be one of the most ancient forms of medicine still practiced. The Ayurvedic preparation *Raudra Rasa* and its derivatives have been widely employed against cancer since the 12th century, but the effect of these traditional formulations on platelet function and signaling has not previously been examined. Here we demonstrate that *Raudra Rasa* and its derivatives significantly reduce thrombin‐induced integrin activation and granule secretion in platelets, as observed by reduced PAC‐1 binding and P‐selectin externalization, respectively. These formulations also inhibited thrombin‐stimulated phosphatidylserine exposure, mitochondrial reactive oxygen species generation, and mitochondrial transmembrane potential in platelets. Consistent with the above, *Raudra Rasa* significantly reduced thrombin‐induced tyrosine phosphorylation of the platelet proteins, as well as phosphorylation of the enzymes AKT and GSK‐3β. In summary, *Raudra Rasa* inhibits agonist‐mediated platelet activation without affecting cell viability, suggesting it may have therapeutic potential as an anti‐platelet/anti‐thrombotic agent.

## Graphical abstract

*Raudra Rasa* derivatives significantly attenuate thrombin‐induced platelet responses, including activation of integrins α_IIb_β_3_, secretion of granule contents, increased mROS/Δψ_m_, and phosphorylation of AKT and its downstream effector GSK‐3β, thus negatively regulating the thrombin‐PAR‐AKT‐GSK‐3β signaling axis in activated platelets. These observations underscore the anti‐platelet effects of *Raudra Rasa* and its derivatives and suggest their potential as anti‐thrombotic agents.

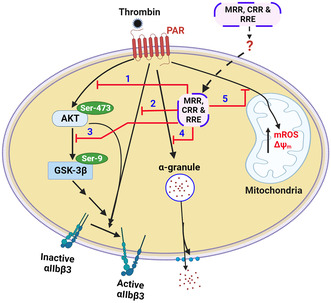

**Abbreviations**
CRR: Classical *Raudra Rasa*
MFI: mean fluorescence intensity
MRR: Modified *Raudra Rasa*
RRE: *Raudra Rasa* extract

Ayurveda is considered one of the most ancient forms of Indian traditional medicine. *Raudra Rasa*, a prominent Ayurvedic preparation, has been used for the treatment of cancer (also known as *arbuda*). Classical *Raudra Rasa* (CRR) (major ingredients: *shadguna kajjali*, decoction of *Piper betel Linn*., *Amaranthus spinosus Linn*., *Boerhaavia diffusa Linn., and Piper longum Linn*.), modified *Raudra Rasa* (MRR) (major ingredients: *hiraka bhasma* or nanoscale diamond, *shadguna rasasindura*, water‐soluble extract of *Piper betel Linn*., *Amaranthus spinosus Linn*., *Boerhaavia diffusa Linn*., and *Piper longum Linn*.) and *Raudra Rasa* extract (RRE) (major ingredients: *Piper betel Linn*., *Amaranthus spinosus Linn*., *Boerhaavia diffusa Linn.,* and *Piper longum Linn*.) have well‐established anti‐cancer properties [1].

Platelets are circulating cells that play a central role in hemostasis and thrombosis. While platelets are instrumental in the control of hemorrhage, their hyperactivity underlies serious pathologies, like ischemic stroke and acute myocardial infarction. Although platelets are enucleate dead‐end cells, unlike their cancer counterparts, emerging evidence from laboratories, including ours, intriguingly suggest several similarities in biology between both the cell types. For example, upon stimulation platelets switch their energy metabolism to Warburg's aerobic glycolysis, similar to cancer cells [2]. Both express developmental morphogens like Sonic hedgehog (Shh) and Notch, which are integral to multiplication of cancer cells and activation of platelets [3, 4, 5]. Small‐molecule modulators of aerobic glycolysis (e.g. dichloroacetate), and Shh and Notch signaling pathways (e.g. vismodegib and DAPT, respectively), that are widely tried as potent anti‐cancer agents, have also been shown to possess strong anti‐platelet/anti‐thrombotic attributes [4, 5]. Previously, it has been shown that Diabet, which is a polyherbal formulation, has potent anti‐platelet activity [6]. However, the studies using *Raudra Rasa* on platelet function remains elusive. Therefore, it is pertinent to ask whether *Raudra Rasa,* having profound anti‐cancer therapeutic benefits, too, have anti‐platelet properties.

In this study we examined the effect of Ayurvedic formulations like CRR, MRR, and RRE on human platelet function. Here, for the first time, we demonstrate significant anti‐platelet properties of these preparations, as revealed from reduced binding of PAC‐1 to surface integrins α_IIb_β_3_ and P‐selectin externalization, decreased mitochondrial transmembrane potential, phosphatidylserine exposure, and mitochondrial reactive oxygen species generation in thrombin‐induced platelets. These formulations, too, attenuated thrombin‐stimulated protein phosphorylation on tyrosine residues, AKT, and GSK‐3β phosphorylation in human platelets. Remarkably, *Raudra Rasa* and its derivatives did not have any cytotoxic effect or impact on platelet viability at the doses employed in the study. Thus, *Raudra Rasa* formulations may be repurposed as potential anti‐platelet therapeutic agents.

## Materials and methods

### Materials

PAC‐1 (# 340507), CD62P (# 550561), and Annexin V (# 640906) antibodies were procured from BD Biosciences (Gurugram, India) and BioLegend (San Diego, CA, USA), respectively. Antibodies against pY99 (#sc‐7020) was from Santa Cruz Biotechnology (Dallas, TX, USA). Antibodies against p‐AKT (Ser473; # 4051), AKT (# 9272), p‐GSK‐3β (Ser9; # 9336), and GSK‐3β (# 12456) were purchased from Cell Signaling Technology (Danvers, MA, USA). Anti‐actin antibody (# A2066), thrombin (# T6884), prostaglandin E_1_ (# P5515) were from Sigma‐Aldrich (Bangalore, India). Collagen (# 385) was purchased from Chrono‐log (Chennai, India). Chemiluminescent HRP substrate (# WBKLS0100), skimmed milk powder (# 70166), and polyvinylidene fluoride membrane (# IPVH00010) were obtained from Millipore (Bangalore, India). MitoTracker Red (# M7512), MitoSOX Red (# M36008), and Cell Viability Assay Kit (# G7570) were procured from Invitrogen and Promega (New Delhi, India), respectively. HRP‐conjugated goat anti‐rabbit IgG (# 114038001A) and goat‐anti‐mouse IgG (# 114068001A) antibodies were from Bangalore Genei (Bangalore, India). Bovine serum albumin (# GRM105) was from HIMEDIA (Maharashtra, India). Stripping buffer (# 21059) was purchased from Thermo Fisher Scientific (Mumbai, India). Tween‐20, CaCl_2_ and other reagents were from Merck (Bangalore, India). Materials employed in the preparation of CRR, MRR, and RRE were purchased at the Faculty of Ayurveda, Institute of Medical Sciences, Banaras Hindu University, Varanasi, as described [1].

### Methods

#### Preparation of CRR, MRR, and RRE

CRR, MRR, and RRE were prepared and validated at the Faculty of Ayurveda, Institute of Medical of Sciences, Banaras Hindu University, Varanasi, as described previously [1]. Briefly, CRR was prepared by using *shadguna kajjali* levigated with the decoction of *Piper betel Linn*., *Amaranthus spinosus Linn*., *Boerhaavia diffusa Linn*., *Piper longum Linn*., and others. MRR was prepared by mixing of hiraka bhasma, *shadguna rasasindura*, water‐soluble extract of *Piper betel Linn*., *Amaranthus spinosus Linn*., *Boerhaavia diffusa Linn*., *Piper longum Linn.,* among others. RRE was prepared from *Piper betel Linn*., *Amaranthus spinosus Linn*., *Boerhaavia diffusa Linn*., *Piper longum Linn*. and others. Detailed methods for the preparation and validation of these formulations were described previously [1].

#### Platelet preparation

Healthy human participants were recruited at the Department of Biochemistry, Institute of Medical Sciences, Banaras Hindu University, and blood was drawn in an acid citrate dextrose (ACD) vial under written informed consent. The study was conducted strictly as per recommendations as approved by the Ethical Committee of the Institute of Medical Sciences, Banaras Hindu University (No. Dean/2019/EC/1683). Platelets were isolated from fresh peripheral venous human blood by differential centrifugation, as described previously [5]. Cells were counted using automated cell counter (Multisizer 4, Beckman Coulter, Mumbai, India) and leukocyte contamination in platelet isolate was found to be less than 0.015%. The study methodologies were conducted according to the standards set by the Declaration of Helsinki.

#### Study of platelet integrin activation

To determine the platelet integrin activation, human platelets were pretreated with either CRR (100, 150, or 200 μg·mL^−1^) or MRR (50, 100, or 200 μg·mL^−1^) or RRE (100, 200 μg·mL^−1^ or 1.25, 2.5 mg·mL^−1^) or vehicle for 30 min at RT, followed by thrombin‐stimulation (0.25 U·mL^−1^, for 5 min at 37 °C). Cells were stained with PAC‐1 (FITC‐labeled) antibody (5% v/v) for 30 min at RT in the dark. After incubation, cells were suspended in sheath fluid and subjected to flow cytometry (FACSCalibur, BD Biosciences). Data were analyzed by cellquest pro Software (Gurugram, India), as described previously [5].

#### Platelet–platelet interaction (aggregation)

Whole blood was pretreated with either vehicle or CRR (200 μg·mL^−1^) or MRR (200 μg·mL^−1^) or RRE (2.5 mg·mL^−1^) for 30 min at RT, followed by addition of collagen (5 μg·mL^−1^). Platelet aggregation/platelet–platelet interaction in whole blood was recorded in a lumi‐aggregometer (Chrono‐log, model 700‐2) as change in electrical resistance (impedance) as a function of time.

#### Study of platelet α‐granules secretion

α‐granules secretion was quantified by surface externalization of P‐selectin. Human platelets were pretreated with either CRR (100, 150, or 200 μg·mL^−1^) or MRR (50, 100, or 200 μg·mL^−1^) or RRE (100, 200 μg·mL^−1^ or 1.25, 2.5 mg·mL^−1^) or vehicle for 30 min at RT, followed by thrombin‐stimulation (0.25 U·mL^−1^, for 5 min at 37 °C). Cells were stained with anti‐CD62P (PE‐labeled) antibody (5% v/v) for 30 min in the dark at RT and analyzed by flow cytometry, as described previously [5].

### Flow cytometric analysis of mitochondrial transmembrane potential (Δψ_m_)

For the determination of Δψ_m_, platelets were pretreated with either CRR (100, 150, or 200 μg·mL^−1^) or MRR (50, 100, or 200 μg·mL^−1^) or RRE (100, 200 μg·mL^−1^ or 1.25, 2.5 mg·mL^−1^) or vehicle for 30 min at RT followed by thrombin‐stimulation (0.25 U·mL^−1^) for 5 min at 37 °C. Cells were stained with MitoTracker Red dye (500 nm) for 45 min in the dark at RT and analyzed by flow cytometry.

### Western blot analysis

Proteins from platelet lysate were separated on 10% sodium dodecyl sulphate‐polyacrylamide gel (SDS/PAGE) gels and transferred onto polyvinylidene difluoride membranes for 20 min at 20 V/1.3 A, as described [7]. Membranes were blocked with blocking buffer (5% skimmed milk or 5% BSA) for 1 h at RT, followed by overnight incubation with specific antibodies (anti‐p‐AKT, 1:1000; anti‐AKT, 1:1000; anti‐p‐GSK‐3β, 1:1000; anti‐GSK‐3β, 1:1000; anti‐pY99, 1:5000; anti‐actin, 1:5000). Membranes were washed with TBST and incubated with HRP‐conjugated secondary antibodies for 1 h at RT. Binding of antibody was determined using enhanced chemiluminescence detection kit (Millipore). p‐AKT‐ and p‐GSK‐3β‐stained membranes were stripped and reprobed with anti‐AKT and anti‐GSK‐3β antibodies, as described [5]. Images were acquired on the UVP BioSpectrum 800 Imaging System and quantified using visionworks ls software (UVP, Upland, CA, USA).

### Phosphatidylserine exposure

Human platelets were pretreated with either CRR (100 or 200 μg·mL^−1^) or MRR (100 or 200 μg·mL^−1^) or RRE (1.25 or 2.5 mg·mL^−1^) or vehicle for 30 min at RT, followed by thrombin‐stimulation (0.25 U·mL^−1^) for 5 min at 37 °C. Cells were stained with annexin V antibody (FITC‐labeled) (5% v/v) in the presence of 5 mm calcium for 30 min at RT in the dark. The 10,000 annexin V positive events were acquired from the gated region and analyzed by flow cytometry.

### Measurement of mitochondrial ROS

Generation of mitochondrial superoxide from human platelets was determined as described [8]. Briefly, platelets pretreated with either CRR (100 or 200 μg·mL^−1^) or MRR (100 μg·mL^−1^) or RRE (100 μg·mL^−1^) or vehicle for 30 min at RT following thrombin‐stimulation (0.25 U·mL^−1^) for 5 min at 37 °C. Platelets were labeled with MitoSOX Red (5 μm) for 15 min in the dark at RT and analyzed by flow cytometry.

### Cell viability assay

Cell viability was determined by employing luciferase‐coupled ATP quantitation assay kit (CellTiter‐Glo viability assay, Promega), which determines the number of viable platelets based on the quantitation of ATP, a measure of metabolically active cells [9]. Human platelets were preincubated with either CRR (100, 150, or 200 μg·mL^−1^) or MRR (50, 100, or 200 μg·mL^−1^) or RRE (100, 200 μg·mL^−1^ or 1.25, 2.5 mg·mL^−1^) or vehicle for 30 min at RT followed by with or without thrombin‐stimulation (0.25 U·mL^−1^) for 5 min at 37 °C. Cells were collected in 96‐well white‐walled microtiter plates, to which an equal volume of CellTiter‐Glo reagent was added and incubated for 10 min at RT. The reagent lyses the cells, generating luminescent signal, which is proportional to the amount of ATP present in the lysate. Generated luminescence signal was measured by multimodal microplate reader (BioTeK model Synergy H1, Santa Clara, CA, USA).

### Statistics

Repeated measures (RM) one‐way ANOVA with Dunnett's multiple comparisons test was used for evaluation of the data and *P* < 0.05 was considered significant. Analysis of all the data was carried out using graphpad prism software (v. 8.4, Santa Clara, CA, USA) and presented as mean ± SEM of at least four independent experiments.

## Results

### CRR, MRR, and RRE impair thrombin‐induced integrin activation in human platelets in a dose‐dependent manner

**Fig. 1:**
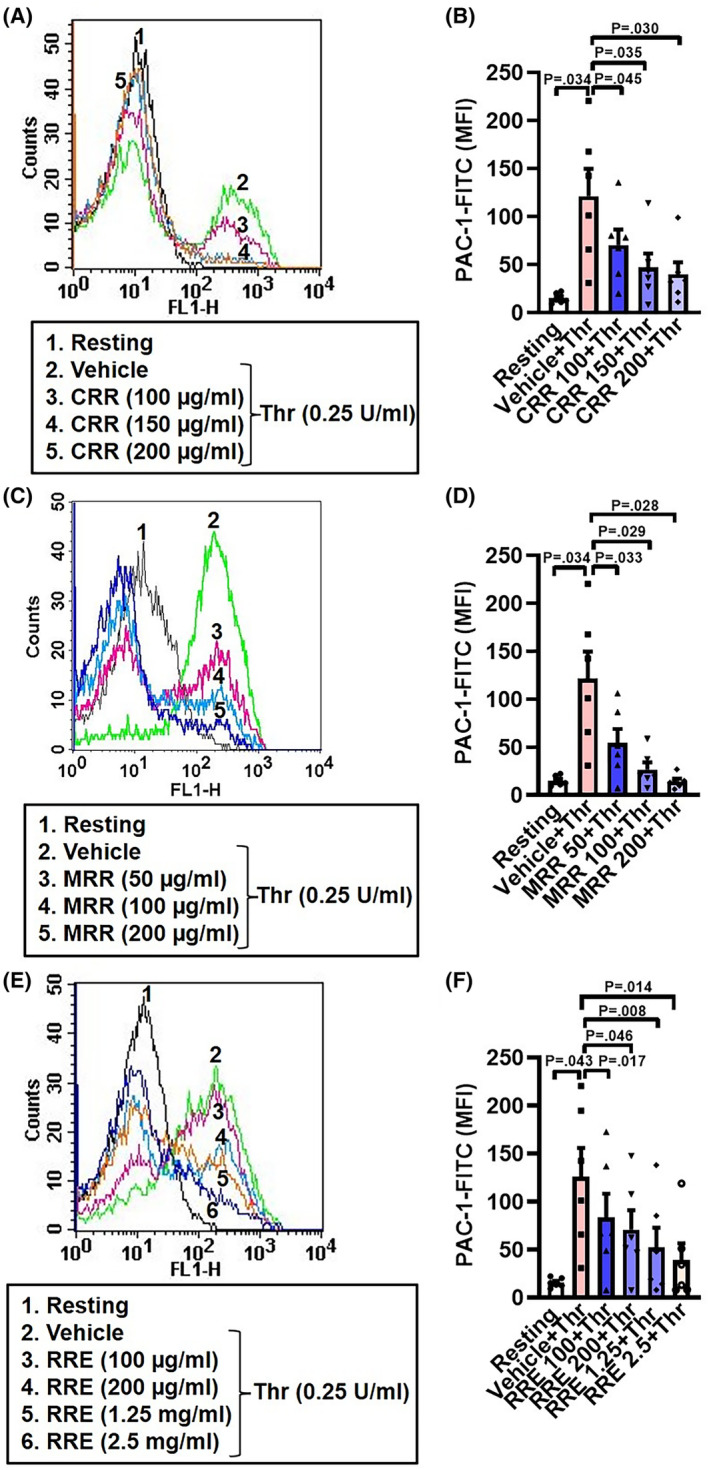
CRR, MRR, and RRE attenuate integrin activation in human platelets in a dose‐dependent manner. A,C,E: histograms showing binding of PAC‐1 to platelets pretreated with either CRR (100–200 μg·mL^−1^) or MRR (50–200 μg·mL^−1^) or RRE (100 μg·mL^−1^ to 2.5 mg·mL^−1^) or vehicle for 30 min at RT followed by stimulation with thrombin (Thr, 0.25 U·mL^−1^) for 5 min at 37 °C, as indicated. B (*n* = 6), D (*n* = 6), and F (*n* = 6), the corresponding bar charts showing mean fluorescence intensity (MFI) of PAC‐1 binding to platelets. Data are presented as mean ± SEM and analyzed by RM one‐way ANOVA with Dunnett's multiple comparisons test.

Conformational switch of integrins α_IIb_β_3_ is a hallmark of platelet activation. In order to examine the effect of CRR, MRR, and RRE on thrombin‐induced integrin activation, we preincubated platelets with increasing doses of either CRR (100, 150, or 200 μg·mL^−1^), MRR (50, 100, or 200 μg·mL^−1^), or RRE (100, 200 μg·mL^−1^, 1.25 or 2.5 mg·mL^−1^) for 30 min at RT, followed by stimulation with thrombin (0.25 U·mL^−1^, for 5 min at 37 °C). Interestingly, we observed that CRR, MRR, and RRE significantly attenuated thrombin‐induced PAC‐1 binding in platelets in a dose‐dependent manner (Fig. 1A–F). Platelet activation ushers conformational switch in surface integrins α_IIb_β_3_ that allows high‐affinity binding of fibrinogen as well as PAC‐1 antibody (that specifically recognizes the conformationally active α_IIb_β_3_), leading to cell–cell aggregate formation. In support of this, CRR (200 μg·mL^−1^), MRR (200 μg·mL^−1^), and RRE (2.5 mg·mL^−1^), too, profoundly retarded collagen‐mediated platelet aggregation/platelet–platelet interaction in whole blood measured from electronic impedance (Fig. S1A,B).

### CRR, MRR, and RRE impair thrombin‐induced P‐selectin externalization in human platelets in a dose‐dependent manner

**Fig. 2:**
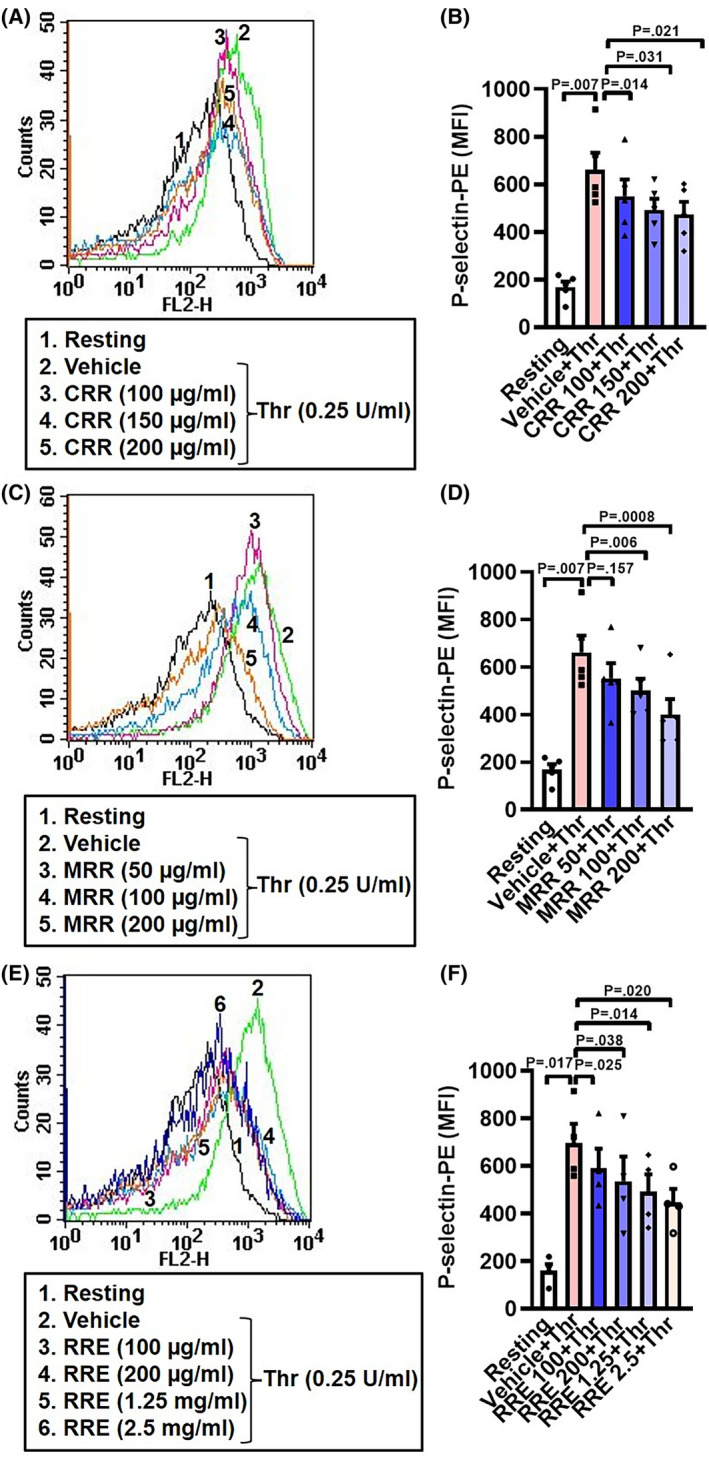
CRR, MRR, and RRE attenuate P‐selectin externalization in human platelets in a dose‐dependent manner. A,C,E: histograms showing binding of P‐selectin antibody (anti‐CD62P) to platelets pretreated with either CRR (100–200 μg·mL^−1^) or MRR (50–200 μg·mL^−1^) or RRE (100 μg·mL^−1^ to 2.5 mg·mL^−1^) or vehicle for 30 min at RT followed by stimulation with thrombin (0.25 U·mL^−1^) for 5 min at 37 °C, as indicated. B (*n* = 5), D (*n* = 5), and F (*n* = 4), the corresponding bar charts showing mean fluorescence intensity (MFI) of P‐selectin antibody binding to platelets. Data are presented as mean ± SEM and analyzed by RM one‐way ANOVA with Dunnett's multiple comparisons test.

Platelet activation leads to secretion of its α‐granule contents with exposure of P‐selectin on the surface membrane. In order to examine the effect of CRR, MRR, and RRE on thrombin‐induced P‐selectin externalization, we preincubated platelets with increasing doses of either CRR, MRR, or RRE for 30 min, followed by stimulation with thrombin. Each of the *Raudra Rasa* formulations significantly attenuated thrombin‐induced P‐selectin externalization in platelets in a dose‐dependent manner (Fig. 2A–F).

### CRR, MRR, and RRE impair thrombin‐induced mitochondrial transmembrane potential (Δψ_m_) in human platelets in a dose‐dependent manner

**Fig. 3:**
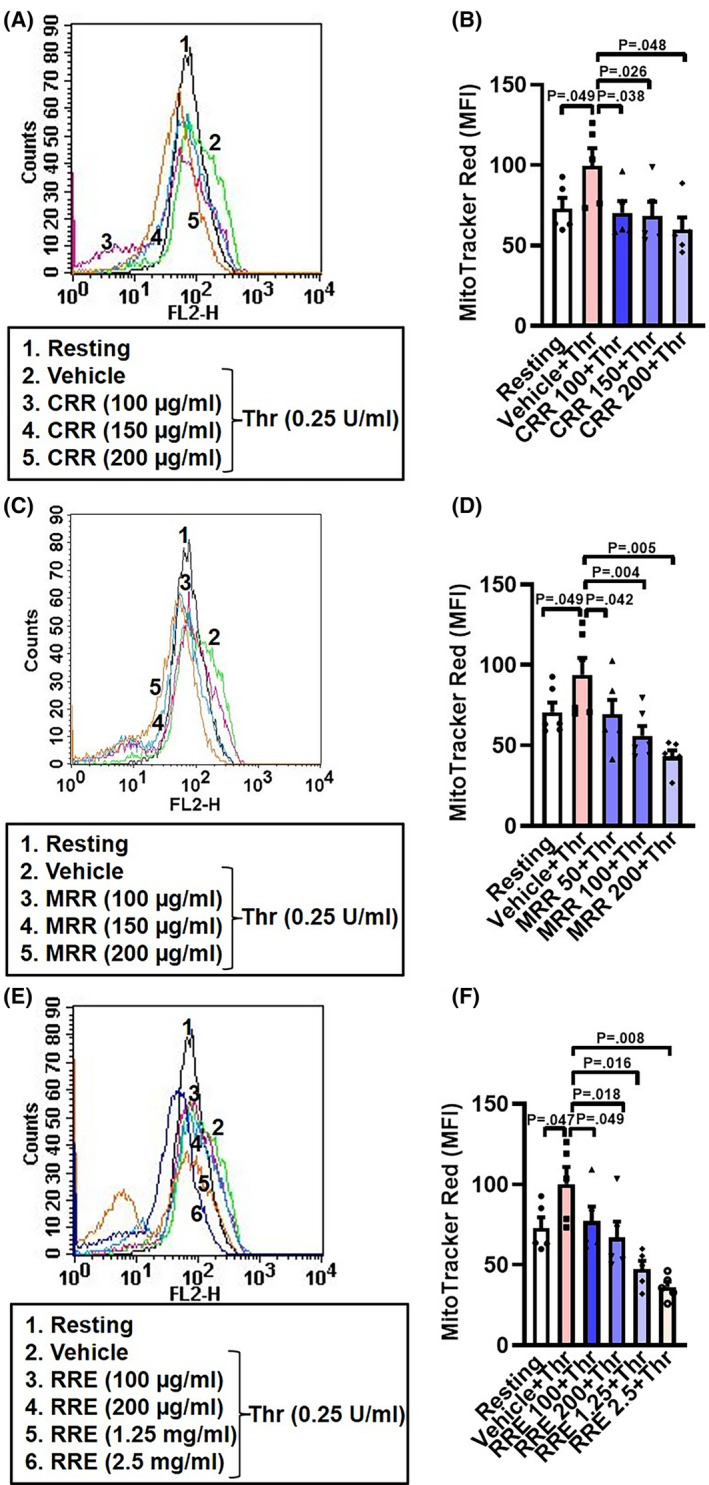
CRR, MRR, and RRE attenuate mitochondrial transmembrane potential in human platelets in a dose‐dependent manner. (A,C,E) histograms showing binding of MitoTracker Red dye to platelets pretreated with either CRR (100–200 μg·mL^−1^) or MRR (50–200 μg·mL^−1^) or RRE (100 μg·mL^−1^ to 2.5 mg·mL^−1^) or vehicle for 30 min at RT followed by stimulation with thrombin (0.25 U·mL^−1^) for 5 min at 37 °C, as indicated. B (*n* = 5), D (*n* = 6), and F (*n* = 5), the corresponding bar charts showing mean fluorescence intensity (MFI) of MitoTracker Red dye binding to platelets. Data are presented as mean ± SEM and analyzed by RM one‐way ANOVA with Dunnett's multiple comparisons test.

Maintenance of Δψ_m_ across platelet inner mitochondrial membrane is reflective of well‐coupled functional mitochondria. Platelets challenged with thrombin were observed to have significantly enhanced Δψ_m_, as studied with MitoTracker Red‐labeled platelets. In order to examine the effect of *Raudra Rasa* derivatives, we preincubated cells with varying doses of formulations as above, followed by stimulation with thrombin. Each of the three preparations significantly attenuated thrombin‐induced mitochondrial transmembrane potential in platelets in a dose‐dependent manner (Fig. 3A–F), suggestive of their critical role in inciting proton leak across the inner membrane that could restrict energy production underlying platelet activity.

### CRR, MRR, and RRE restrain thrombin‐induced AKT and GSK‐3β phosphorylation and expression of tyrosine phosphoproteome in human platelets

**Fig. 4:**
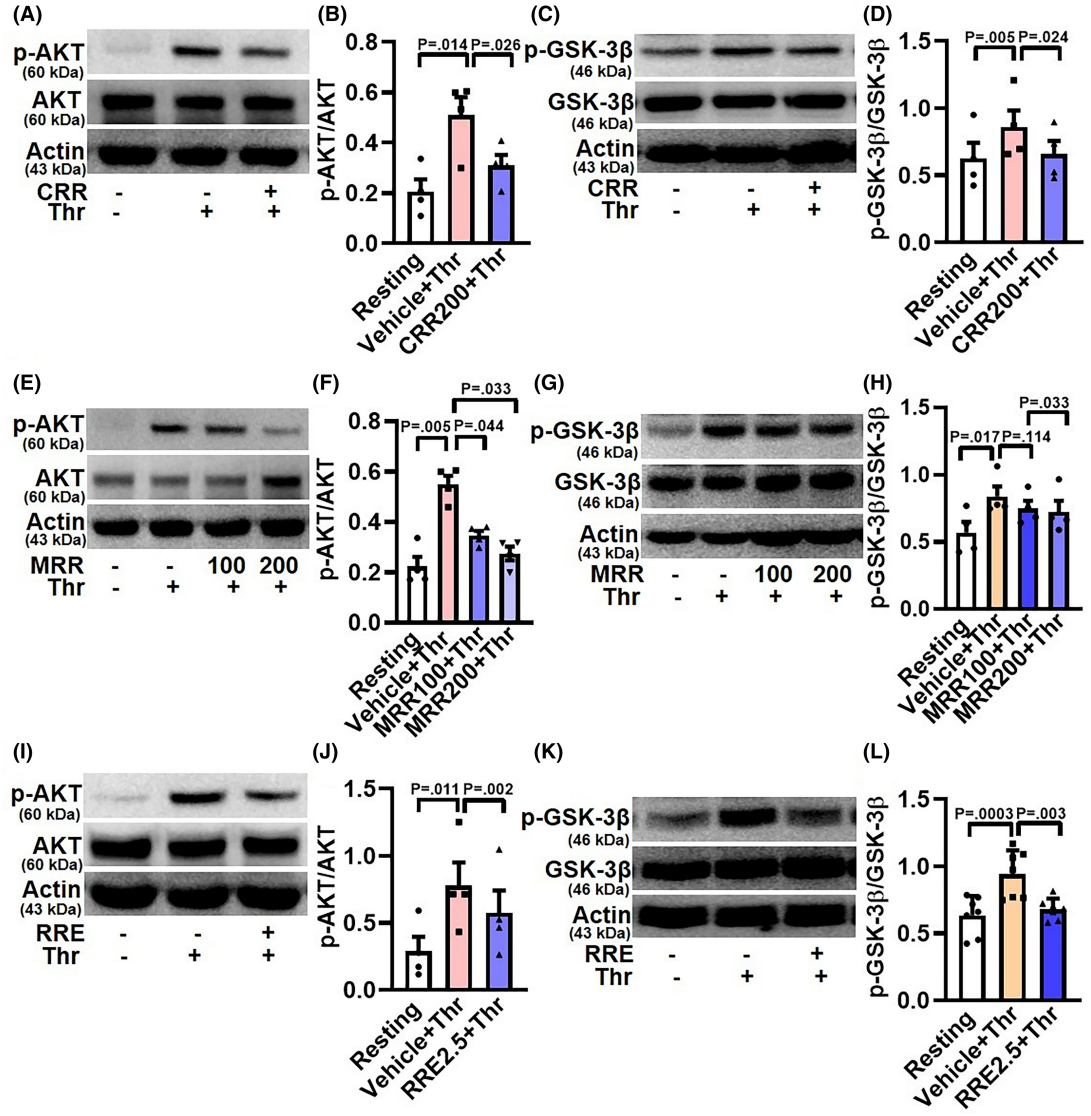
Effect of CRR, MRR, and RRE on thrombin‐induced AKT and GSK‐3β phosphorylation in human platelets. Platelets were pretreated with either CRR (200 μg·mL^−1^) or MRR (100 and 200 μg·mL^−1^) or RRE (2.5 mg·mL^−1^) or vehicle for 30 min at RT followed by stimulation with thrombin (0.25 U·mL^−1^) for 5 min at 37 °C, as indicated. (A,E,I) Western analysis showing expression of p‐AKT. (C,G,K) Western analysis showing expression of p‐GSK‐3β. B (*n* = 4), F (*n* = 4), and J (*n* = 4), the corresponding densitometric analyses of p‐AKT normalized with AKT. D (*n* = 4), H (*n* = 4), and L (*n* = 7), the corresponding densitometric analyses of p‐GSK‐3β normalized with GSK‐3β. Data are presented as mean ± SEM and analyzed by RM one‐way ANOVA with Dunnett's multiple comparisons test.

Agonist‐induced platelet stimulation incites phosphorylation and activation of AKT kinase, the downstream effector of phosphoinositide 3‐kinase (PI3K) [10, 11]. Activation of AKT consequently leads to the phosphorylation of glycogen synthase kinase (GSK)‐3β, its downstream effector molecule [12]. Thrombin (0.25 U·mL^−1^) elicited robust phosphorylation of AKT, which was significantly abrogated upon pretreatment of cells with either CRR (200 μg·mL^−1^), MRR (100 or 200 μg·mL^−1^), or RRE (2.5 mg·mL^−1^) (by 39.21% for CRR; 37.16% and 49.91%, respectively, for MRR; and 26.19% for RRE) (Fig. 4A,B,E,F,I,J). Thrombin‐stimulation, too, significantly upregulated phosphorylation of GSK‐3β similar to AKT. Pretreatment of platelets with either CRR (200 μg·mL^−1^), MRR (100 or 200 μg·mL^−1^), or RRE (2.5 mg·mL^−1^) significantly attenuated thrombin‐stimulated GSK‐3β phosphorylation (by 23.19% for CRR; 10.74% and 13.48%, respectively, for MRR; and 27.66% for RRE) in platelets (Fig. 4C,D,G,H,K,L). Thus, the above observations underscore the contribution the AKT‐GSK‐3β signaling axis in thrombin‐challenged platelets exposed to these Ayurvedic formulations.

**Fig. 5:**
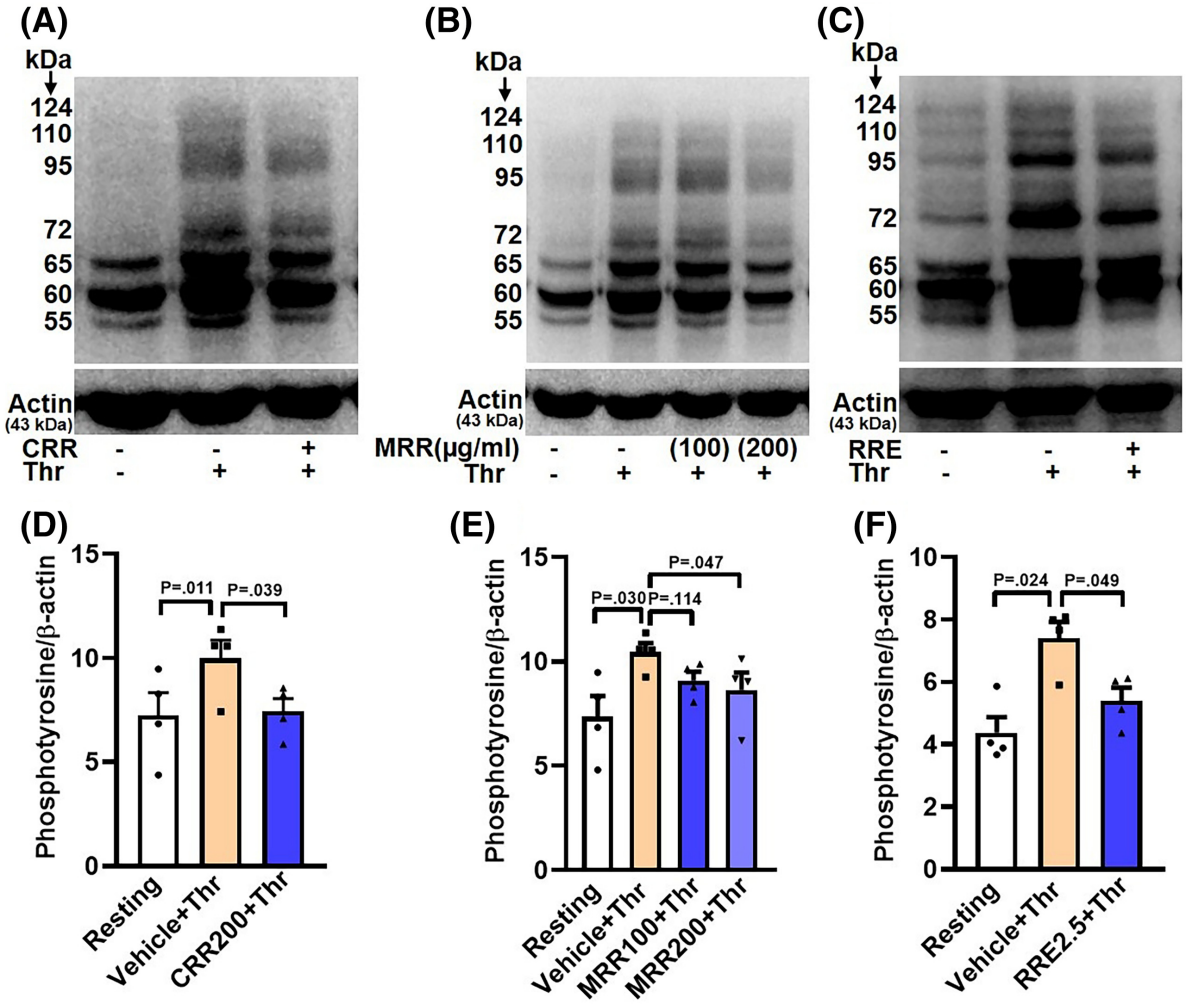
Effect of CRR, MRR, and RRE on thrombin‐induced tyrosine phosphoproteome in human platelets. (A–C) Western analysis of platelets preincubated with either CRR (200 μg·mL^−1^) or MRR (100 or 200 μg·mL^−1^) or RRE (2.5 mg·mL^−1^) or vehicle for 30 min at RT, followed by stimulation with thrombin (0.25 U·mL^−1^) for 5 min at 37 °C, as indicated. D (*n* = 4), E (*n* = 4), and F (*n* = 4), the corresponding densitometric analyses of phosphotyrosine normalized with β‐Actin. Data are presented as mean ± SEM and analyzed by RM one‐way ANOVA with Dunnett's multiple comparisons test.

Platelet hyperactivity is associated with phosphorylation of multiple cytosolic proteins on tyrosine residues due to activation of *Src* kinases [13]. Pretreatment of platelets with *Raudra Rasa* preparations brought about a remarkable drop in expression of tyrosine‐phosphorylated proteins (molecular masses 55, 60, 65, 72, 95, 110, and 124 kDa) compared to the vehicle‐treated counterparts (Fig. 5A–F).

### CRR, MRR, and RRE attenuate thrombin‐induced phosphatidylserine (PS) exposure in human platelets

**Fig. 6:**
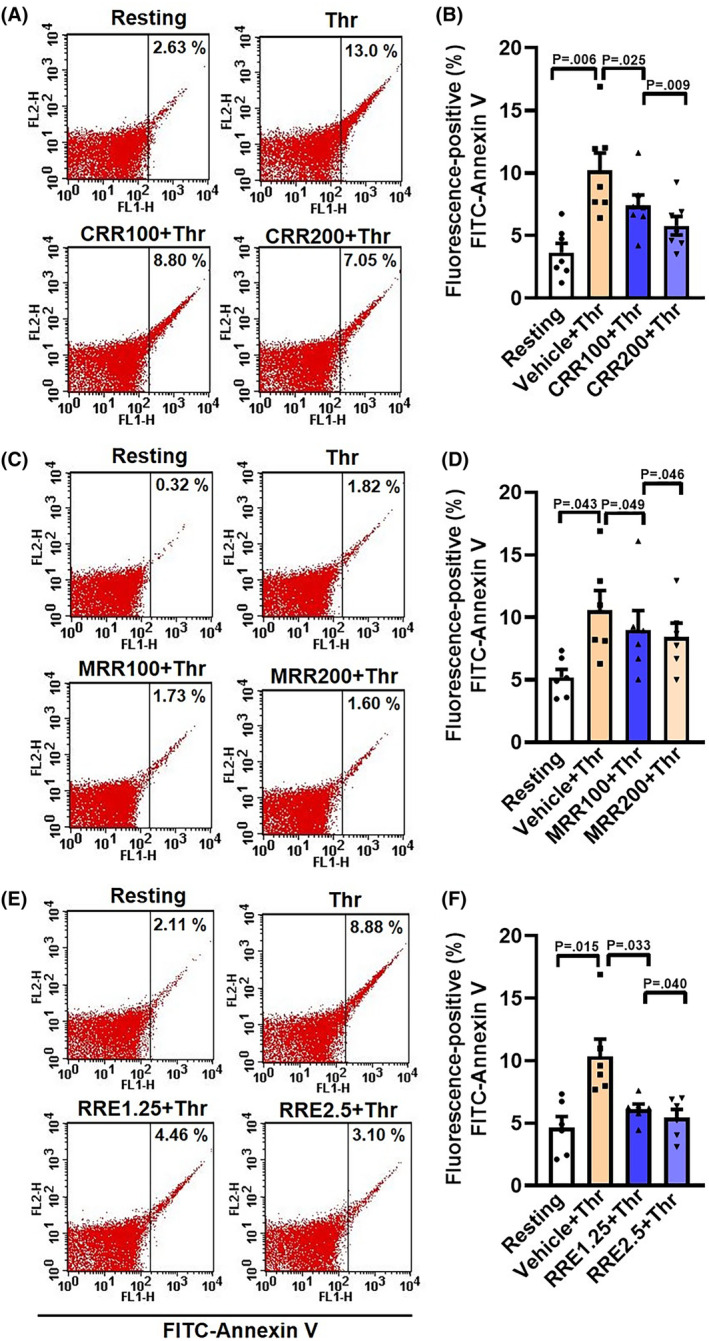
CRR, MRR, and RRE attenuate thrombin‐induced phosphatidylserine exposure in human platelets. A,C,E: dotplot showing binding of annexin V to platelets pretreated with either CRR (100 or 200 μg·mL^−1^) or MRR (100 and 200 μg·mL^−1^) or RRE (1.25 or 2.5 mg·mL^−1^) or vehicle for 30 min at RT, followed by stimulation with thrombin (0.25 U·mL^−1^) for 5 min at 37 °C, as indicated. B (*n* = 7), D (*n* = 6), and F (*n* = 6), the corresponding bar charts showing mean per cent of annexin V binding to platelets. Data are presented as mean ± SEM and analyzed by RM one‐way ANOVA with Dunnett's multiple comparisons test.

Phosphatidylserine translocates from the inner to outer leaflet of the plasma membrane in agonist‐challenged platelets, thus generating a procoagulant surface with high affinity towards annexin V [14]. Exposure to thrombin raised PS expression on the platelet surface, as evident from increased annexin V binding, which was significantly retarded upon pretreatment with increasing concentrations of either CRR (100 or 200 μg·mL^−1^), MRR (100 or 200 μg·mL^−1^), or RRE (1.25 or 2.5 mg·mL^−1^) (by 27.62% and 43.48% for CRR; 15.05% and 20.26% for MRR; and 42.13% and 48.01% for RRE, respectively) (Fig. 6A–F). The above observations are consistent with an anti‐thrombogenic effect of *Raudra Rasa* and its derivatives.

### CRR and MRR, but not RRE, attenuate thrombin‐induced mitochondrial reactive oxygen species (mROS) generation in human platelets

**Fig. 7:**
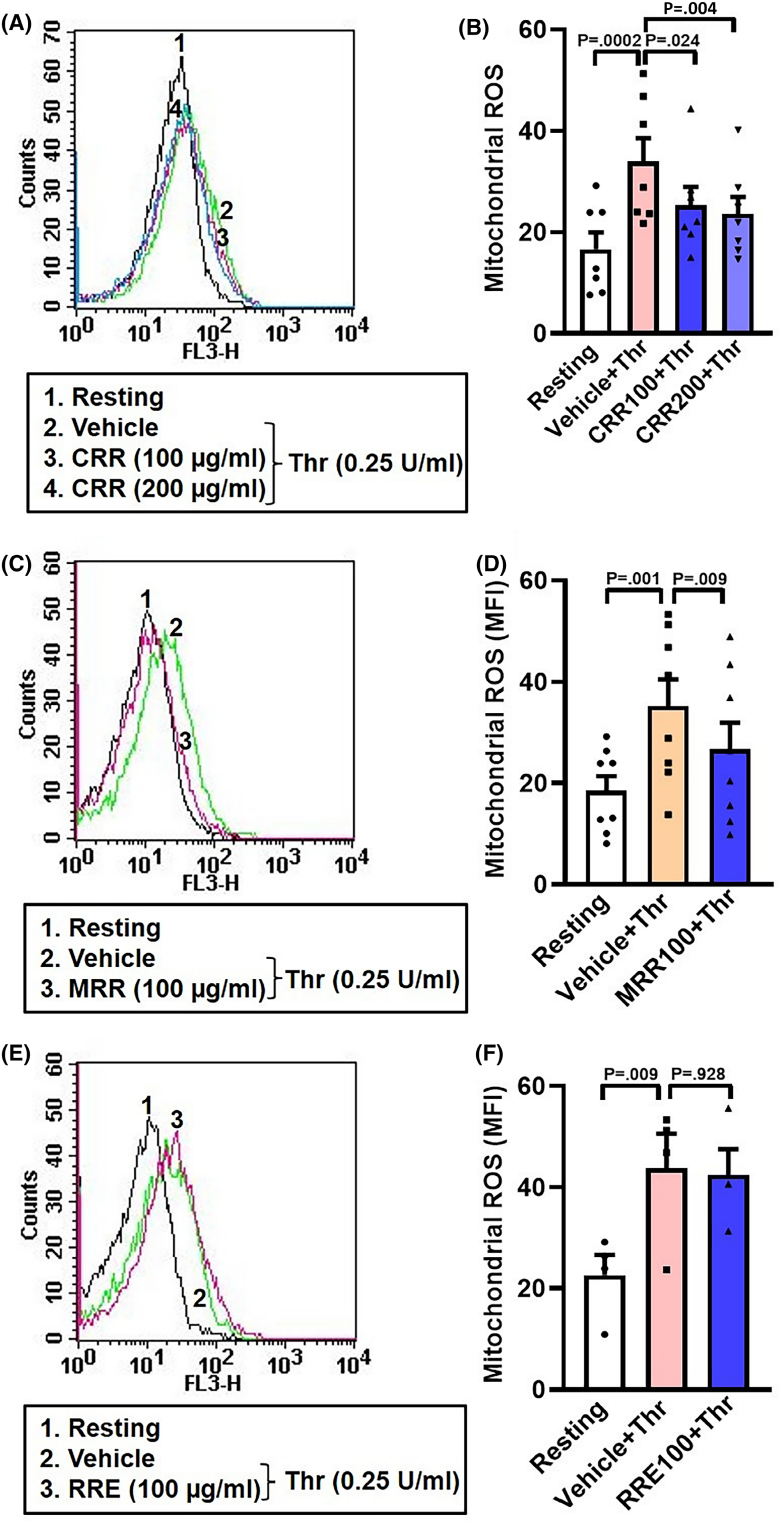
Effect of pretreatment with CRR, MRR, and RRE on thrombin‐induced mitochondrial ROS generation in human platelets. A,C,E: histograms of MitoSOX Red dye binding to platelets pretreated with either CRR (100 or 200 μg·mL^−1^) or MRR (100 μg·mL^−1^) or RRE (100 μg·mL^−1^) or vehicle for 30 min at RT, followed by stimulation with thrombin (0.25 U·mL^−1^) for 5 min at 37 °C, as indicated. B (*n* = 7), D (*n* = 8), and F (*n* = 4), the corresponding bar charts showing mean fluorescence intensity (MFI) of MitoSOX Red dye binding to platelets. Data are presented as mean ± SEM and analyzed by RM one‐way ANOVA with Dunnett's multiple comparisons test.

Reactive oxygen species (ROS) are considered as key players for platelet hyperactivity [2, 8] and mitochondria are also a significant source of ROS in platelets [8]. In order to determine the effect of CRR, MRR, and RRE on mROS generation in thrombin‐stimulated platelets, we preincubated cells with either CRR (100 or 200 μg·mL^−1^) or MRR (100 μg·mL^−1^) or RRE (100 μg·mL^−1^) or vehicle for 30 min at RT following thrombin‐stimulation (0.25 U·mL^−1^) for 5 min at 37 °C. As expected, thrombin evoked a rise in mROS, which was significantly mitigated by CRR and MRR (Fig. 7A–F). However, RRE was unable to elicit such responses at the doses employed. ROS being an important mediator of platelet activation [2, 15], the above observations implicate ROS in anti‐platelet effects of *Raudra Rasa* and its formulations.

### Effect of CRR, MRR, and RRE on cell viability in thrombin‐stimulated human platelets

**Fig. 8:**
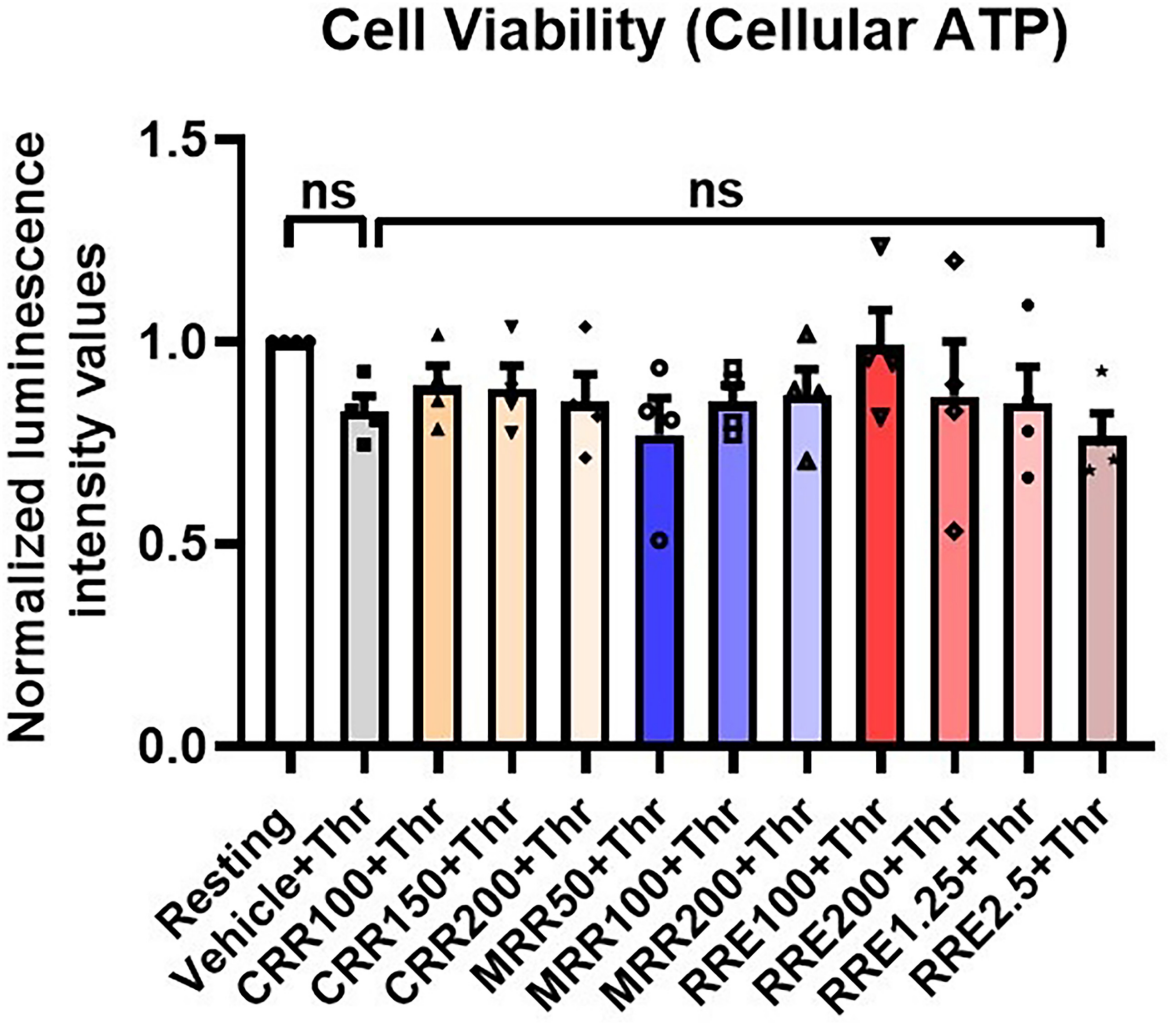
Effect of CRR, MRR, and RRE on human platelets viability. Bar diagram representing cell viability of platelets preincubated with either vehicle or CRR (100–200 μg·mL^−1^) or MRR (50–200 μg·mL^−1^) or RRE (100 μg·mL^−1^ to 2.5 mg·mL^−1^) for 30 min at RT, followed by stimulation with thrombin (0.25 U·mL^−1^) for 5 min at 37 °C, as indicated (*n* = 4). Data are presented as mean ± SEM. ns (nonsignificant) compared to either resting or thrombin‐treated platelets and analyzed by RM one‐way ANOVA with Dunnett's multiple comparisons test.

As *Raudra Rasa* and its modifications considerably attenuated mROS generation and PS externalization in human platelets, we next asked whether these drugs affect platelet viability. Remarkably, when platelets were incubated with increasing doses of either CRR (100, 150, or 200 μg·mL^−1^) or MRR (50, 100, or 200 μg·mL^−1^) or RRE (100 or 200 μg·mL^−1^ or 1.25 or 2.5 mg·mL^−1^) or vehicle for 30 min at RT following thrombin‐stimulation (0.25 U·mL^−1^) for 5 min at 37 °C had no significant effect on cell viability, as revealed from the level of cellular ATP. Thus, the *Raudra Rasa* formulations inhibit agonist‐induced platelet activation and restrict thrombogenicity without affecting platelet viability or inducing any cytotoxic effect (Fig. 8).

## Discussion

Ayurveda is considered one of the most ancient forms of Indian traditional medicine. *Raudra Rasa* has been used in Ayurvedic pharmacies for the treatment of cancer since the 12th century. However, the role of these traditional formulations on platelet function and signaling has remained unexplored. Platelets play a key role in hemostasis and thrombosis that can instigate serious pathologies like ischemic stroke and myocardial infarction. Emerging evidence from laboratories, including ours, suggest uncanny similarities between the biology of platelets and cancer cells. For example, activated platelets change their energy metabolism to Warburg's aerobic glycolysis, similar to cancer cells [2]. Both cell types express developmental morphogens like Sonic hedgehog (Shh) and Notch, which are integral to multiplication of cancer cells and activation of platelets [3, 4, 5]. Small‐molecule modulators of aerobic glycolysis (e.g. dichloroacetate), as well as Shh and Notch signaling pathways (e.g. vismodegib and DAPT, respectively), that are widely tried as potent anti‐cancer agents, have also been shown to be endowed with significant anti‐platelet/anti‐thrombotic attributes [4, 5].

In this report we demonstrated that *Raudra Rasa* and its different derivatives significantly attenuated thrombin‐induced platelet responses that include activation of surface integrins α_IIb_β_3_ and secretion of granule contents. Agonist‐induced platelet activation provokes phosphorylation and activation of ser‐thr kinase AKT [10, 11]. Activated AKT leads to the phosphorylation and activation of its downstream effector ser‐thr kinase GSK‐3β, which is regulated upon its phosphorylation at Ser‐9 [12]. These Ayurvedic formulations significantly retarded thrombin‐stimulated phosphorylation of AKT (Ser‐473) in platelets. It has been previously reported that agonist‐induced phosphorylation of GSK‐3β is dependent on AKT activity [12]. We, too, observed that Ayurvedic formulations significantly precluded thrombin‐stimulated phosphorylation of GSK‐3β in platelets. Thrombin binds with PAR1/PAR4 receptors on the surface of human platelets, leading to AKT phosphorylation, which, in turn, incites phosphorylation of GSK‐3β at Ser‐9 [12]. The above observations underscored the pivotal role of these formulations in negative regulation of the PAR‐AKT‐GSK‐3β signaling axis in thrombin‐stimulated platelets.

Platelets are known to abundantly express *Src* family protein tyrosine kinases such as *Src*, *Lyn,* and *Fyn*, as well as other tyrosine kinases like Syk, whose activities are considerably upregulated upon platelet stimulation through integrin inside‐out and outside‐in signaling events [16, 17, 18]. In this report we determined the role of these formulations on platelet tyrosine phosphoproteome. It was striking to observe that the above formulations brought about a remarkable drop in tyrosine phosphoproteome in thrombin‐stimulated platelets. Thus, the results overall underscored the fact that *Raudra Rasa* formulations significantly attenuated the prothrombotic phenotype of agonist‐challenged platelets by modulating the AKT‐GSK‐3β axis, as well as the expression of tyrosine phosphoproteome, leading to a drop in platelet aggregation. Notably, these preparations dissipated mitochondrial transmembrane potential (Δψ_m_) in thrombin‐stimulated platelets, leading to severely compromised ATP generation and consequent loss in platelet reactivity [19]. Decreased PS exposure in the presence of these formulations restrained the platelets from switching to a procoagulant phenotype. All three formulations of *Raudra Rasa* have an almost similar inhibitory effect on platelet function. Although the mechanism of action of these Ayurvedic formulations remain an area for future research, it is safe to posit that they adversely affect the agonist–receptor interaction, thus constraining inside‐out signaling in thrombin‐stimulated platelets. Taken together, the above observations underscore significant anti‐platelet effects of *Raudra Rasa* and its derivatives without affecting platelet viability or inducing any cytotoxic effect, and hence could be employed as potential anti‐platelet/anti‐thrombotic agents.

## Conflict of interest

The authors declare no conflicts of interest.

### Peer review

The peer review history for this article is available at https://www.webofscience.com/api/gateway/wos/peer‐review/10.1002/2211‐5463.13713.

## Author contributions

SNC: Conceptualization, Formal analysis, Validation, Investigation, Visualization, Methodology, Writing – original draft, Writing – review and editing; VS: Investigation, Methodology, Formal analysis, Validation; ME: Investigation, Methodology; MKD: Resources (prepared and validated *Raudra Rasa* formulations); NJ: Resources (prepared and validated *Raudra Rasa* formulations); DD: Conceptualization, Supervision, Funding acquisition, Validation, Investigation, Visualization, Project administration, Writing – original draft, Writing – review and editing.

## Supporting information


**Fig. S1.** CRR, MRR and RRE attenuate collagen‐mediated platelet aggregation in whole blood. A, whole blood was pretreated with either CRR (tracing 2) or MRR (tracing 3) or RRE (tracing 4) or vehicle (tracing 1) for 30 min at RT, followed by addition of collagen (5 μg·mL^−1^) for 5 min at 37 °C. Platelet aggregation was recorded as change in electrical resistance (impedance) as a function of time. B, corresponding bar chart shows collagen‐induced mean platelet aggregation in whole blood (*n* = 4). Data are presented as mean ± SEM and analyzed by RM one‐way ANOVA with Dunnett's multiple comparisons test.

## Data Availability

All the data needed to draw the conclusions are available either in the article or the supporting information.
