# Supplementary figures and images for: Ayurvedic preparations of Raudra Rasa inhibit agonist‐mediated platelet activation and restrict thrombogenicity without affecting cell viability
Source: FEBS Open Bio. 2023 Nov 21;13(12):2342–55. doi: 10.1002/2211-5463.13713 (PMC10699108; doi:10.1002/2211-5463.13713)

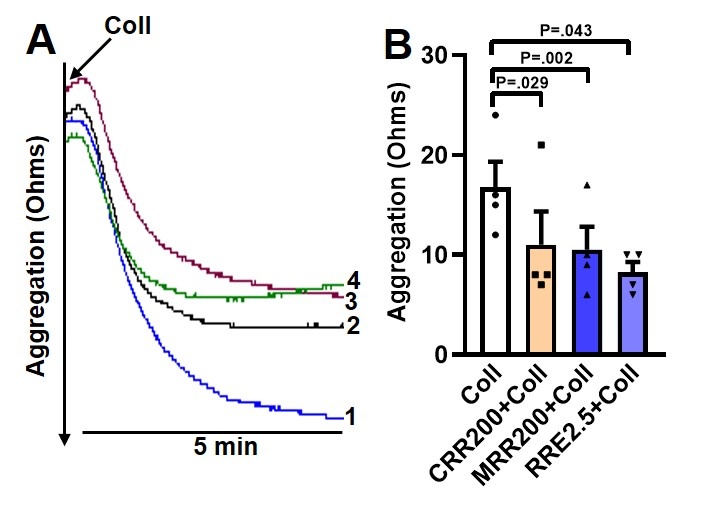

Supplement: Supplementary file 1 — Fig. S1. CRR, MRR and RRE attenuate collagen‐mediated platelet aggregation in whole blood. A, whole blood was pretreated with either CRR (tracing 2) or MRR (tracing 3) or RRE (tracing 4) or vehicle (tracing 1) for 30 min at RT, followed by addition of collagen (5 μg·mL−1) for 5 min at 37 °C. Platelet aggregation was recorded as change in electrical resistance (impedance) as a function of time. B, corresponding bar chart shows collagen‐induced mean platelet aggregation in whole blood (n = 4). Data are presented as mean ± SEM and analyzed by RM one‐way ANOVA with Dunnett's multiple comparisons test. [file FEB4-13-2342-s001.jpg]
